# Exploring the Therapeutic Potential of Ketamine and Psilocybin in Comparison to Current Treatment Regimens for Treatment-Resistant Depression, Mood Disorders, and Post-traumatic Stress Disorder in the Pediatric Population: A Narrative Review

**DOI:** 10.7759/cureus.90425

**Published:** 2025-08-18

**Authors:** Brett Hughes, Soz Mirza, Manasi Ponamala, James Sagaser, Riley Paredes, Naomi Hematillake, Chandni Tailor, Rahim Khan, Sudhakar Pemminati

**Affiliations:** 1 Department of Biomedical Education, California Health Sciences University College of Osteopathic Medicine, Clovis, USA

**Keywords:** ketamine, mood disorders, post traumatic stress disorder (ptsd), psilocybin, treatment-resistant depression

## Abstract

The stresses of the Coronavirus Disease of 2019 (COVID-19) pandemic highlighted the burden of psychiatric disorders within the pediatric population, revealing a pre-existing need for rapid-onset therapies that have since driven efforts to expand effective therapeutic interventions. In this narrative review, we utilized the Preferred Reporting Items for Systematic reviews and Meta-Analysis (PRISMA) guidelines to direct our report and study selection. We explored the current-state efficacy and therapeutic potential of ketamine and psilocybin in comparison to current treatment regimens for pediatric non-psychotic disorders, including Treatment-Resistant Depression (TRD), mood disorders like anxiety and bipolar disorder, and Post-Traumatic Stress Disorder (PTSD). We chose these pediatric disorders to eliminate concerns regarding reality orientation and the use of dissociative and/or psychedelic medicines in patients who are experiencing symptoms of psychosis. Also, we briefly discuss ketamine’s more widely accepted utilization by medical providers as a pediatric anesthetic, and how this gives credence to further evaluation of ketamine’s multifaceted indications in pediatric psychiatry. Recent studies have shed light on the involvement of glutamate pathways in the pathophysiology of TRD, mood disorders, and PTSD, and both ketamine, an N-methyl-D-aspartate (NMDA) receptor antagonist, and psilocybin, a 5-hydroxytryptamine receptor 2A (5-HT2A) agonist, have emerged as promising options due to their ability to augment glutamate release. Ketamine’s use for pediatric TRD demonstrated rapid-onset relief for signs and symptoms of depression in children and adolescents, and psilocybin also decreased symptoms in patients with longstanding or refractory depression. Ketamine has been well tolerated and exhibited symptom improvements for youth with mood disorders such as anxiety and bipolar depression, while psilocybin showed promise in fostering emotional processing. In youth suffering from PTSD, ketamine-assisted psychotherapy (KAP) brought about decreases in PTSD symptom severity, though outcomes varied across populations. Psilocybin enhanced neural plasticity, allowing patients to revisit and reframe memories under therapeutic guidance, especially for those with complex or treatment-resistant PTSD. Ethical considerations are involved in the use of dissociative and hallucinogenic therapies like ketamine and psilocybin in the pediatric population, and we explore some ethical issues regarding their use. Further research exploring specific brain locations and mechanisms of action underlying glutamate modulation by ketamine and psilocybin, and the subsequent rapid-acting relief of psychiatric symptoms offered by these substances, could pave the way for innovative treatments targeting pediatric mental health disorders.

## Introduction and background

Pediatric focus and need-based rationale

Ketamine and psilocybin are two emerging innovative therapies for treatment-resistant psychiatric disorders, offering potential rapid-onset treatment opportunities to pediatric patients who have not experienced symptom resolution with conventional treatments. Currently, in the pediatric population, there exists an urgent clinical need for fast-acting therapies that can provide prompt symptom relief, especially during psychiatric crises such as acute suicidality [1]. However, ketamine and psilocybin use are not currently a common standard of practice as fast-acting therapies, though pediatric and adult studies have shown rapid and sustained positive effects on patients with Treatment-Resistant Depression (TRD), mood disorders like anxiety and bipolar disorder, and Post-Traumatic Stress Disorder (PTSD) [1,2]. Our aim in this inquiry is to explore if, and how, this urgent clinical need for fast-acting therapy might be met by the therapeutic potential ketamine and/or psilocybin offer.

Ketamine context and mechanism

**Figure 1:**
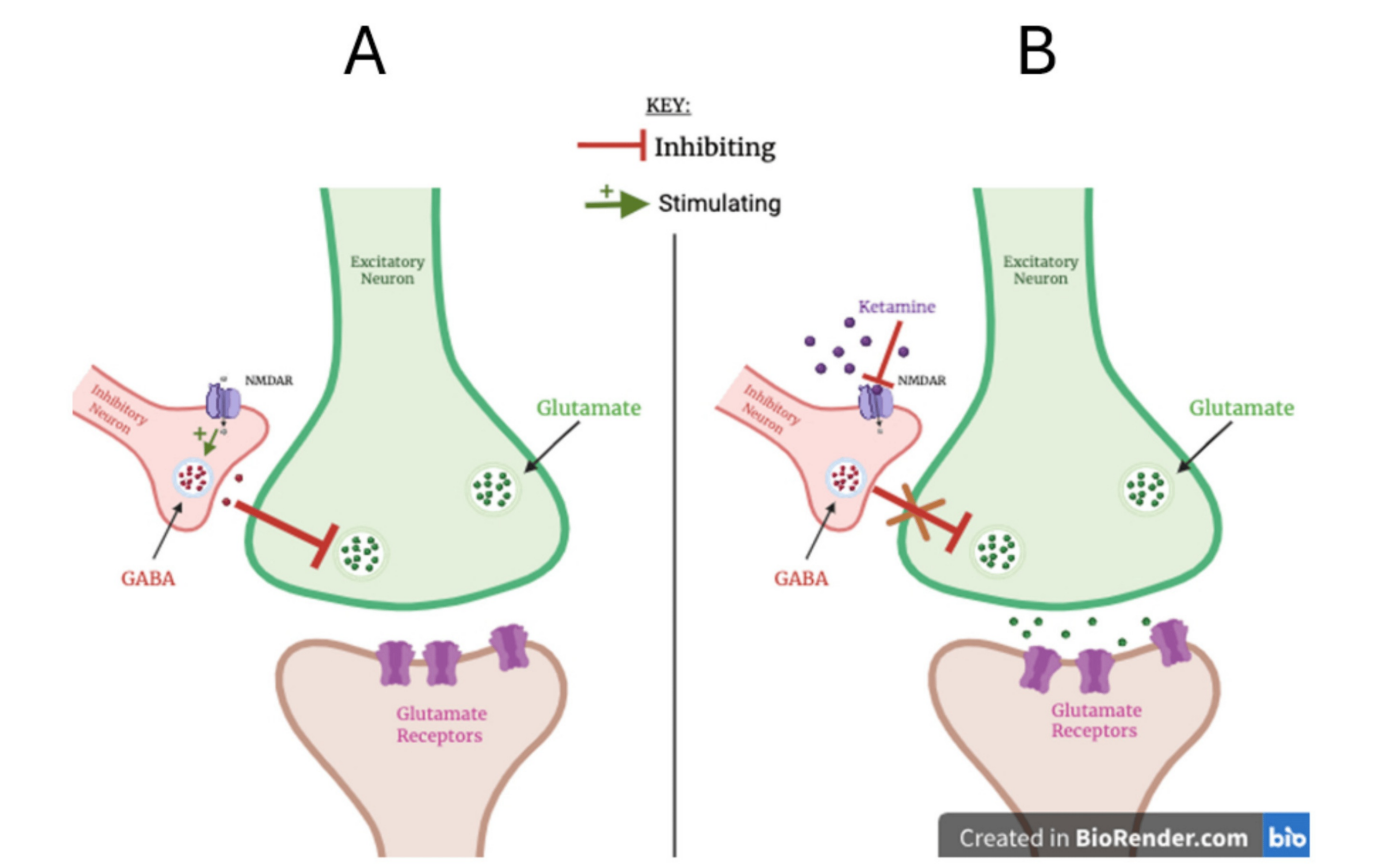
A: Neurotransmission; B: Ketamine's mechanism of action Image credit: Manasi Ponamala; NMDAR: N-methyl-D-aspartate receptor; GABA: gamma-aminobutyric acid

Initially developed in the 1960s as an anesthetic, ketamine has evolved into a multifaceted therapeutic agent used in multiple medical disciplines, such as surgery and psychiatry [3]. As a dissociative anesthetic, ketamine functions primarily through the non-competitive antagonism of the N-methyl-D-aspartate receptor (NMDA, a type of ionotropic glutamate receptor that is a ligand-gated ion channel found throughout the central nervous system), thus augmenting glutamate signaling in the brain and inhibiting the release of gamma-aminobutyric acid (GABA), as Figure 1 displays [4].

As an anesthetic target, glutamate’s role as the primary excitatory neurotransmitter affecting wakefulness and consciousness is well known, and ketamine's pharmacokinetics and clinical utility in pediatric populations as an anesthetic have been extensively studied [5]. Now, an accumulating body of evidence is also showing glutamatergic dysfunction plays a key role in many psychiatric disorders [6]. For example, both postmortem and clinical neuroimaging studies have indicated that patients with depression have decreased glutamate and glutamate-receptor levels in the anterior cingulate cortex and prefrontal cortex (specifically the dorsolateral region), and studies have indicated the severity of glutamatergic dysregulation may be correlated with the severity of psychiatric illness [6]. Genome-wide association studies have also provided genetic evidence that glutamate signaling abnormalities are involved in the pathophysiology of bipolar disorder [6]. Similarly, accumulating evidence points to abnormal glutamatergic function and/or dysfunction in glutamate neurotransmission as a characteristic feature of stress-related psychiatric disorders, including PTSD [7]. Therefore, glutamate modulation underpins not only ketamine’s anesthetic properties, but also ketamine’s potential to alleviate depressive symptoms, regulate mood, and heal trauma, including individuals with treatment-resistant conditions, when administered at subanesthetic dosages [8]. Pre-clinical, clinical, and animal studies’ evidence has displayed that the anterior cingulate cortex is involved in ketamine’s antidepressant effects, alongside the interconnected lateral habenula, striatum, and hippocampus, though a precise mechanism remains unclear [9].

While much of the research on ketamine’s psychiatric applications has focused on adults, and studies involving pediatrics are limited, its potential benefit for pediatric populations is becoming a subject of interest, and the existing evidence explored here provides a foundation for further evaluation regarding its use in the pediatric demographic [10]. When administered in controlled clinical settings, ketamine produces rapid-acting and sustained antidepressant effects in children, allowing some pediatric patients to experience relief within hours or days of treatment [4, 11]. This sharply contrasts to some more traditional antidepressants, such as selective serotonin reuptake inhibitors (SSRIs), which have delayed efficacy and can take weeks to have full effect [1]. Also, children and adolescents who have not responded to conventional treatments, or those with treatment-resistant conditions, may benefit from ketamine’s rapid action and unique mechanism of action, positioning it as a potential alternative for these individuals [8]. Concerns about ketamine’s safety profile, potential for misuse, and long-term effects are relevant in pediatric populations, and while more rigorous studies are required to establish its safety and efficacy for psychiatric applications in younger patients, ketamine’s ability to prevent emergence agitation and its promising role in mood disorder management suggest significant clinical utility [8, 12]. Hence, ketamine has significant potential to fill the clinical need for rapid-acting psychiatric therapy in pediatrics.

Psilocybin context and mechanism

Another novel therapeutic agent worth investigating for the pediatric psychiatry population is psilocybin. As a naturally occurring molecule found in specific types of mushrooms predominantly found in Mexico, South America, and subtropical regions of the United States, psilocybin is a hallucinogenic compound and one of the first psychoactive substances discovered [2]. It is currently being studied as a promising treatment for TRD, mood disorders like anxiety and bipolar disorders, and PTSD in adults [2]. Historically associated with recreational drug use and popularized in the United States also in the 1960s, psilocybin is now being reevaluated as an official medical treatment for certain psychiatric illnesses [13]. Studies have shown psilocybin to be significantly effective when used as an adjunctive treatment to psychotherapy [14]. With measured treatment, clinical trials have demonstrated a decrease in symptoms of depression, and significantly reduced Montgomery-Åsberg Depression Rating Scale (MADRS) scores [15]. When correlated with current adolescent and young adult case studies, these results indicate potential regarding psilocybin’s benefit for the pediatric population [16, 17].

**Figure 2:**
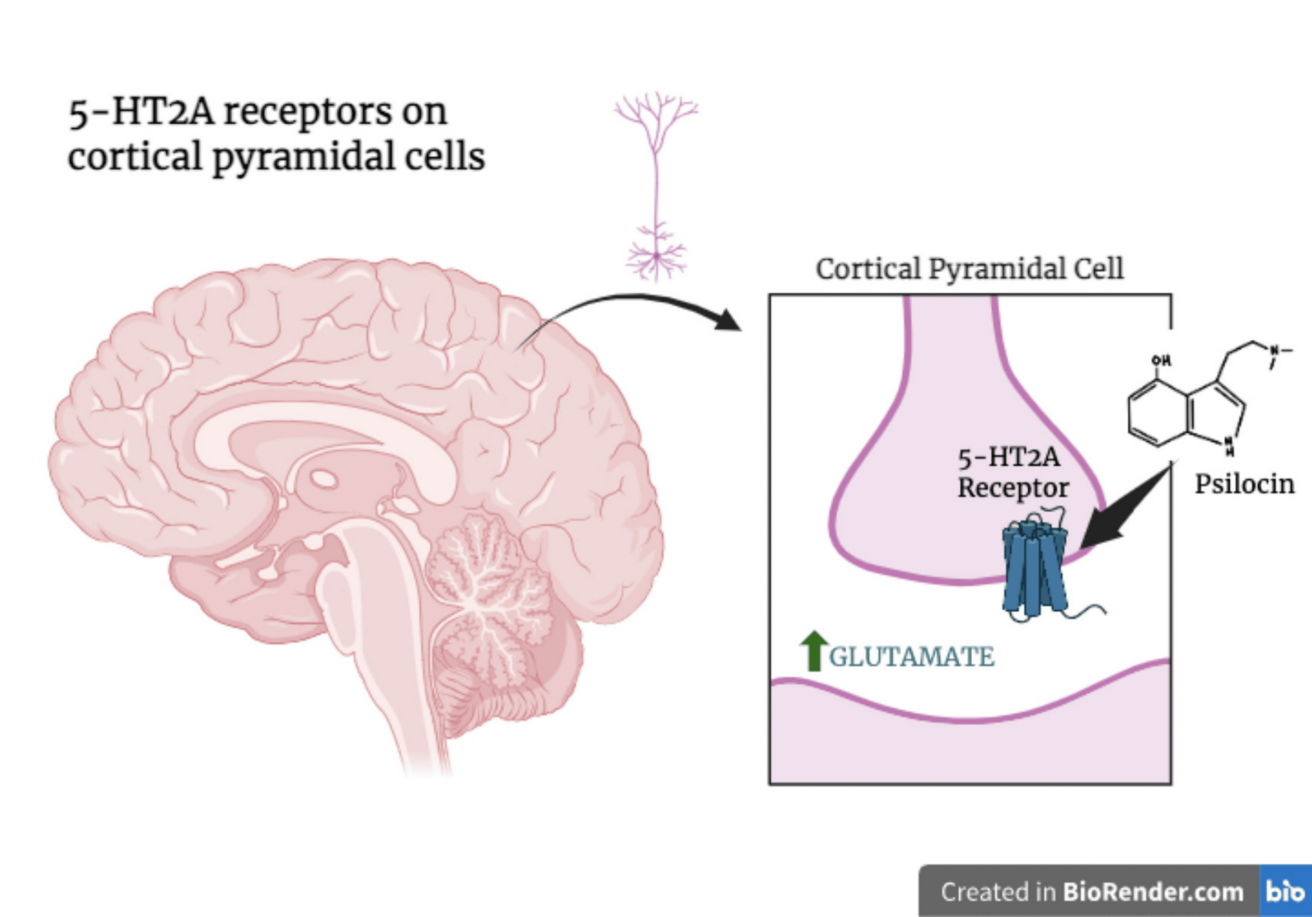
Mechanism of action for psilocin on cortical pyramidal cells Image credit: Manasi Ponamala; 5-HT2A: 5-hydroxytryptamine receptor 2A

Psilocybin falls under the indoleamine category of psychedelics, and its official chemical name is 4-phosphoryloxy-N, N-dimethyltryptamine (4-PO-DMT) [2]. Psilocybin is a prodrug that is dephosphorylated via alkaline phosphatase into an active compound called psilocin (4-OH-dimethyltryptamine), which is a non-selective serotonin 5-hydroxytryptamine receptor 2A agonist (5-HT2AR) agonist [18]. Psilocybin’s clinically relevant effects are credited to its affinity for 5-HT2 receptor subtypes [19]. The 5-HT2 family of serotonin receptors comprises three subtypes (5-HT2A, 5-HT2B, and 5-HT2C), which are G protein-coupled receptors (GPCRs) that possess different distributions, functions, and pharmacological properties, and are biologically naturally activated by serotonin, a key neurotransmitter in mood regulation and perception [20]. The 5-HT2A receptor is primarily expressed in the central nervous system (CNS), with high concentrations in the cerebral cortex, where it is involved in cognition, mood, and psychotropic effects [20]. Psilocin’s psychoactive effects are attributed to the fact that it activates 5-HT2A receptors, specifically located on cortical pyramidal cells, which leads to glutamate release, as depicted in Figure 2 [19]. This glutamate modulation, like with ketamine, positions psilocybin as a potential therapeutic agent with possible beneficial, accelerated effects for pediatric psychiatric patients.

Despite their therapeutic potential, drugs targeting the 5-HT2 receptor subtypes must be carefully evaluated to avoid adverse effects [21]. For example, 5-HT2C inverse agonists offer promise in treating mood disorders, but their discontinuation has a greater likelihood of leading to withdrawal [21]. Understanding the distinct roles of these receptors and their involvement in complex neuropsychiatric conditions highlights the importance of receptor subtype selectivity in developing safe and effective treatments, which is particularly pertinent in the case of psilocybin [22].

Psilocybin’s psychoactive effects are also associated with increased GABA, leading to increased sensory input to the brain [19]. Concerns about side effects, misuse, and long-term safety underscore the need for carefully designed clinical trials, particularly in pediatric populations where the risk-benefit balance must be thoroughly assessed [8,12]. By building on the growing body of evidence, future research will be crucial in determining the optimal role and dosages for both ketamine and psilocybin in pediatric psychiatry, ensuring that their benefits can be realized safely and effectively for all patients.

## Review

Methods

For this narrative review, PubMed, Springer Nature Link, clinicaltrials.gov, Google Scholar, and Embase databases and registries were accessed to identify relevant literature on the therapeutic use of ketamine and psilocybin in pediatric and adolescent populations. The following search terms were used: “ketamine in children,” OR “psilocybin for adolescents,” OR “psychedelic therapy for mood disorders,” OR “ketamine-assisted psychotherapy in adolescents,” OR “psilocybin in treatment-resistant depression,” OR “safety of ketamine in pediatrics,” OR “ketamine and psilocybin for PTSD,” OR “psychedelic-assisted therapy in children.” The search was limited to clinical trials, systematic reviews, meta-analyses, and observational studies published in peer-reviewed journals from 2008 to 2024. The last search date was May 3rd, 2025. Utilizing Preferred Reporting Items for Systematic reviews and Meta-Analysis (PRISMA) guidelines to direct our study selection for this narrative review, we identified and screened records from these searches to facilitate the selection of pertinent reports and studies [23, 24].

Inclusion criteria were studies investigating the safety, efficacy, and mechanisms of ketamine and psilocybin treatments in pediatric and adolescent populations, specifically for TRD, mood disorders like anxiety and bipolar disorders, and PTSD. We excluded studies that (1) were not available in English, (2) were not in the given time frame, (3) lacked clear outcomes, and (4) did not have available full-text articles.

The selected articles were reviewed for data on the therapeutic outcomes, including efficacy in reducing depressive symptoms, improving mood, and addressing anxiety and suicidality. We also reviewed safety data, particularly concerning side effects, adverse events, and the long-term impacts of treatment. Studies focusing on ketamine’s use for TRD, mood disorders like anxiety and bipolar disorders, and PTSD in children and adolescents were prioritized.

**Figure 3:**
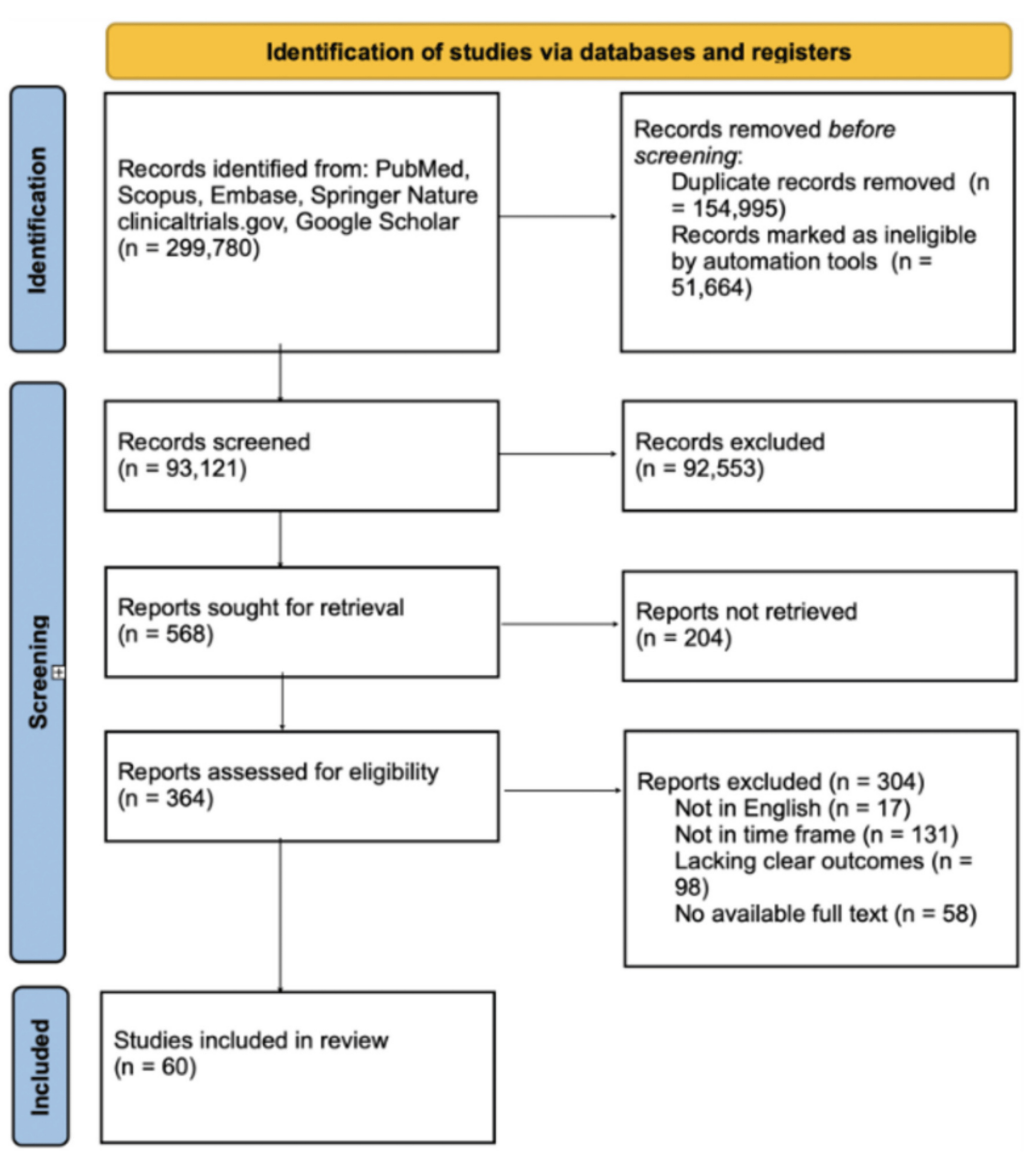
PRISMA flow chart of inclusion and exclusion criteria PRISMA: Preferred Reporting Items for Systematic Reviews and Meta-Analyses

To ensure thoroughness, manual selection was performed to identify any additional relevant studies that may have been missed during the electronic search. Duplicates were removed, and articles were selected for full-text review based on their alignment with the topic and methodological quality (Figure 3).

Current/comparative treatments

While the administration of ketamine and psilocybin as potential treatment modalities for pediatric psychiatric disorders is still early in utilization, the use of ketamine as a dissociative anesthetic is more commonly accepted in the medical community. With neuropharmacological parallels between ketamine and psilocybin, we believe it is important to first review the more established anesthetic uses of these treatments before discussing our focus on non-psychosis psychiatric disorders in the pediatric population. Also, with the emerging nature of both ketamine and psilocybin’s use for pediatric psychiatric treatment, some adult studies with parallel treatment information have been referenced for purposes of correlating these pharmacotherapies’ treatment potential in pediatric TRD, mood disorders, and PTSD.

Anesthesia

Ketamine clinical evidence and comparative efficacy: Ketamine's pharmacokinetics and clinical utility in pediatric populations have been extensively studied when it is used in procedural sedation and anesthesia [5]. Herd et al. found ketamine to be highly safe and effective for procedural sedation in children, with a half-time for equilibration between the effect and central compartments of 11 seconds (95% CI: 0.07-20 seconds) and a half maximal effective concentration (EC50) for arousal at 0.52 mg/L (90% CI: 0.22-1.17 mg/L) [25]. In comparison, alternative anesthetics like propofol and sevoflurane offer distinct anesthetic profiles but present certain limitations. While propofol and sevoflurane provide rapid induction and recovery, it is associated with significant decreases in mean arterial pressure (66 ± 6.77 mmHg for propofol vs. 72.64 ± 7.98 mmHg for sevoflurane, P = 0.0026), limiting their utility in pediatric patients requiring cardiovascular stability [26]. Sevoflurane facilitates faster endotracheal tube extubating times (12.6 ± 5.1 minutes for sevoflurane vs. 13.7 ± 3.6 minutes for propofol, P = 0.194) but is linked to a higher incidence of emergence agitation, affecting 37.4% of children compared to 12.83% with propofol (OR = 4.99, P < 0.00001) [27]. Notably, propofol administration at the end of sevoflurane anesthesia significantly reduces Pediatric Anesthesia Emergence Delirium (PAED) scores from 14.41 ± 2.59 to 9.83 ± 3.51 (P = 0.694), mitigating postoperative complications [28]. These findings underscore ketamine's distinct advantage in providing hemodynamic stability, reliable sedation, and minimal respiratory depression in pediatric patients, thus contributing to ketamine’s increasing acceptability in the medical community as an anesthetic.

Psilocybin clinical evidence and comparative efficacy: Alongside psychiatry, emerging research has drawn parallels between ketamine and psilocybin in their applications within neuropharmacology. Both substances can modulate glutamate signaling, and studies suggest that psilocybin, like ketamine, demonstrates rapid-onset effects, though it operates via 5-HT2A receptor activation rather than NMDA receptor antagonism [19]. Both agents still require careful monitoring for transient dissociative or perceptual effects, and hence their complementary mechanisms and therapeutic outcomes underscore the potentially expanding role of psychedelic anesthetics in modern medicine [2]. Further research is needed before psilocybin can be considered for anesthetic use. The findings are summarized in Table 1.

**Table 1:** Comparative therapies used for anesthesia NMDA: N-methyl-D-aspartate; ICP: Intracranial pressure; GABA-A: Gamma-aminobutyric acid A receptor; SpO2: Saturation of peripheral oxygen

Author/Year/Country	Study Population	Mechanism of Action	Benefits	Adverse Effects	Contraindications/ Cautions
Bali, 2022, India [5]	Pediatric population n = 20,911	Ketamine: NMDA receptor antagonist	Maintains respiratory drive and hemodynamic stability	Transient tachycardia, laryngospasm in infants (rare)	Infants less than 3 months old
Wojtas, 2022, Poland [19]	Preclinical studies in rats	Psilocybin: Modulation of glutamate and serotonin receptors	Insights into neurobiological mechanisms; potential translational benefits	Behavioral disruptions in animal models	Extrapolation limitations from animal to human
Herd, 2008, New Zealand [25]	Pediatric patients n = 43	Ketamine: NMDA receptor antagonist	Rapid onset of anesthesia, predictable mean arousal time	Small potential of raised ICP	Predisposition to laryngospasm or apnea, previous psychotic illness
Patel, 2013, India [26]	Pediatric patients n = 50	Propofol: GABA-A agonist	Intraoperative and postoperative cardiovascular stability	Minor reduction in SpO₂	Respiratory precautions
Zhao, 2022, China [27]	Pediatric patients less than 14 years old n = 1,550	Sevoflurane: GABA-A receptor agonist	Opioid-sparing effect delays the need for rescue analgesia	Pain on injection	Requires intravenous access

Treatment-Resistant Depression (TRD)

Ketamine clinical evidence and comparative efficacy: Ketamine’s role in treating pediatric TRD has been shown to provide encouraging rapid symptom relief, especially for intravenous ketamine, the R-enantiomer of ketamine [29]. During the past few decades, ketamine has become the focus of extensive research, particularly for its rapid-acting antidepressant effects, which contrast sharply with the delayed efficacy of traditional antidepressants like selective serotonin reuptake inhibitors (SSRIs) [1]. A randomized controlled trial (RCT) showed intravenous ketamine significantly reduced depressive symptoms in adolescents (ages 13-17) compared to midazolam, with a mean difference in MADRS scores of 8.69 at 24 hours post-treatment (SD = 15.08, 95% CI = -16.72 to -0.65, p < 0.05), and 76% of participants responded within three days, compared to 35% for midazolam [11]. These effects persisted for 14 days post-treatment when compared to an active placebo, underscoring ketamine’s sustained efficacy in children [11]. Another placebo-controlled trial evaluating esketamine, the S-enantiomer of ketamine, found that adolescents (ages 13-18) who received intravenous esketamine, when compared to midazolam, similarly had reduced depressive symptoms with a mean difference in MARDS scores of 6.5 at six days post-treatment (SD = 11.2, p = 0.004) [30].

In comparing the route of administration for ketamine, one systematic review of pediatric patients with TRD found that while intranasal ketamine was shown to be rapidly effective and well-tolerated despite its precise efficacy being unknown [31], another larger systematic review found intravenous ketamine was nearly three times more effective than intranasal ketamine, with superior response (RR = 3.01 vs. 1.38) and remission rates (RR = 3.70 vs. 1.47) [32]. Subcutaneous racemic ketamine has also been shown to be safe and effective in treating adult TRD over a four-week treatment period, and is an area for further study in pediatrics [33]. Bali et al. report that ketamine exhibits a bioavailability of 90% when administered intramuscularly as well, compared to only 20% when administered orally, making the intramuscular route more effective for inpatient psychiatric clinical use if intravenous infusion is not an option, though parallel studies in children are needed [5]. Adolescents treated with ketamine experienced a 42.5% reduction in depressive symptoms based on Children’s Depression Rating Scale-Revised (CDRS-R) scores, with 38% achieving a clinical response [34]. This evidence also pointed to a dose-response relationship, which indicates a research area of opportunity for further study to identify optimal ketamine dosing for treating pediatric TRD [34]. Finally, Kishimoto et al. highlighted ketamine’s rapid onset of action, with effects beginning within 40 minutes post-infusion, peaking at 24 hours, and lasting up to 10-12 days [35]. Alongside providing increased glutamate modulation, ketamine also induces an increase in molecules that regulate neuroplasticity, which are correlated with these rapid antidepressant effects, and could be beneficial for adolescents with impaired neurogenesis (see ethics discussion below for further inquiry) [36]. These findings highlight ketamine’s rapid, sustained, and clinically significant antidepressant effects, with tolerable side effects, positioning it as a novel treatment option in managing pediatric TRD [35].

Psilocybin clinical evidence and comparative efficacy: Psilocybin shares notable parallels with ketamine in addressing TRD, albeit through distinct mechanisms, and the results of adult studies point to potential benefits for adolescents. Psilocybin exerts its effects through 5-HT receptor activation, enhancing serotonergic activity and promoting structural and functional brain changes [10, 19]. Psilocybin-assisted therapy (PAT) has demonstrated significant efficacy in reducing depressive symptoms, particularly in patients with long-standing or refractory depression [13]. The therapeutic benefits of psilocybin appear to be enhanced when combined with psychotherapy, indicating that it may be most effective in a guided, therapeutic context [37]. In RCTs, psilocybin produced rapid and sustained improvements in mood, with 71% of patients showing marked depressive symptom reduction at six weeks post-treatment, a result comparable to ketamine's 7-10 day efficacy window but with fewer repeat dosing requirements [2]. Furthermore, psilocybin's ability to foster emotional processing and reduce amygdala reactivity complements ketamine's effects on synaptic plasticity, highlighting their potential for synergistic or alternative use in treatment for TRD, possibly for adolescents struggling with such emotional processing [17, 38]. Professor Carhart-Harris at UCSF used functional magnetic resonance imaging (fMRI), cerebral blood flow measurements, and other modes of analysis to show how psilocybin reduced the symptoms of depression in all the participants of the study who had TRD [39]. The reduction of depressive symptoms was correlated through measuring the cerebral blood flow (CBF) to certain regions within the brain [39]. The CBF measured in patients’ amygdala post-treatment with psilocybin showed a significant reduction, which correlated with a reduction in depressive symptoms [2,39]. If these adult studies inspire replication studies in the adolescent population with TRD, it may point to psilocybin as a therapeutic possibility. The findings are summarized in Table 2, and ketamine route, dosing, and administration comparison across Pediatric Treatment Resistant Disorders is summarized in Table 3.

**Table 2:** Comparative therapies used for Treatment Resistant Depression NMDA: N-methyl-D-aspartate; 5-HT2A: 5-hydroxytryptamine receptor 2A; MDD: Major Depressive Disorder; GRID-HAMD: GRID-Hamilton Depression Rating Scale; QIDS-SR: Quick Inventory of Depressive Symptomatology-Self Rated; BP: Blood pressure; HR: Heart rate; MADRS: Montgomery–Åsberg Depression Rating Scale; ASD: Autism Spectrum Disorder; GABA: Gamma-aminobutyric acid; C-SSRS: Columbia Suicide Severity Rating Scale; SpO2: Saturation of peripheral oxygen; CDRS-R: Children's Depression Rating Scale-Revised; TRD: Treatment-Resistant Depression; SUD: Substance Use Disorder; ID: Intellectual disability.

Author/ Year/ Country	Study Population	Mechanism of Action	Benefits	Adverse Effects	Contraindications / Caution
Bali, 2022, India [5]	Narrative review in pediatric patients	Ketamine: NMDA receptor antagonist	Intramuscular route, faster absorption in children	Transient tachycardia and hypotension	Infants less than 3 months due to laryngospasm potential
Barber, 2022, USA [10]	General adult population in psychedelic psychotherapy studies n=178	Psilocybin: 5-HT2A receptor activation	Treats MDD, anxiety, and trauma-related disorders	Transient anxiety, hallucinations, emotional lability	History of psychosis; lack of professional guidance
Dwyer, 2021, USA [11]	13–17-year-old adolescents with MDD n=17	Ketamine: NMDA receptor antagonist	Significant reduction in MADRS with intravenous route	Transient dissociative symptoms, increased BP and HR	History of psychotic disorders, mania, ASD; active suicidal or homicidal ideation
Dwyer, 2021, USA [11]	13–17-year-old adolescents with MDD n=17	Midazolam (control) GABA receptor agonist	Sedation without antidepressant effects	Central nervous system depression	
Davis, 2021, USA [13]	Randomized clinical trial on adult patients with MDD n=24	Psilocybin: 5-HT2A receptor activation	Significant reduction in GRID-HAMD and QIDS-SR scores	Transient elevated HR and BP, headache	Uncontrolled cardiovascular conditions, no personal nor family history of psychotic or bipolar disorders
Zhou, 2023, China [30]	13–18-year-old adolescents with MDD and suicidal ideations n=54	Esketamine (R-ketamine): NMDA receptor antagonist	Significant reduction in C-SSRS Ideation and Intensity scores, reduction in MARDS scores via intravenous route	Nausea, dissociation, sedation, dry mouth, headache, dizziness	Current presence of substance abuse, history of primary psychotic disorder, nonpsychiatric neurological disorder
Meshkat, 2022, Canada & USA [31]	Systematic review of pediatric patients N=45	Ketamine: NMDA receptor antagonist	Safe and effective in treating pediatric depression symptoms, though intranasal efficacy unknown	Self-limiting dissociative symptoms, transient BP, HR, and SpO₂ changes, nausea, dysphoria, nystagmus in one patient	History of psychotic disorders, mania
Cullen, 2018, USA [34]	14–18-year-old adolescents with TRD n=13	Ketamine: NMDA receptor antagonist	Reduction in CDRS-R scores and depressive symptoms through intravenous administration	Transient dissociative symptoms and BP changes, nausea, dysphoria	Current SUD, history of a primary psychotic disorder, ASD, ID, non-psychiatric neurological disorder
Carhart-Harris, 2017, UK [39]	Adult patients with TRD n=19	Psilocybin: 5-HT2A receptor activation; emotional processing	Reduction in amygdala reactivity; enhanced emotional and mood regulation	Potential dysphoric experiences, transient anxiety	Limited dosing studies; careful monitoring needed

**Table 3:** Ketamine route, dosing, and administration comparison across Pediatric Treatment Resistant Disorders MDD: Major Depressive Disorder

Author/ Year/ Country	Study Population	Ketamine Route of Administration	Ketamine Dosing Range	Ketamine Frequency of Administration
Dwyer, 2021, USA [11]	13–17-year-old adolescents with MDD n=17	Intravenous	0.5 mg/kg R-Ketamine infusion over 40 minutes	One time, alternated with Midazolam (0.045 mg/kg) two weeks later
Zhou, 2023, China [30]	13–18-year-old adolescents with MDD and suicidal ideations n=54	Intravenous	0.25 mg/kg S-Ketamine infusion over 40 minutes	Three times over 5 days, compared to Midazolam (0.02 mg/kg)
Meshkat, 2022, Canada & USA [31]	Systematic review of pediatric patients N=45	Intranasal	0.1 mL sprays of 50-200 mg/mL of R-Ketamine per spray, peak doses ranged from 20 to 360 mg per administration	Once every 2-5 days (mean 3.0 ± 0.6 days) over a course of 3 months to 6.5 years

Mood Disorders

Ketamine clinical evidence and comparative efficacy: In mood disorders such as severe anxiety and bipolar depression, ketamine has shown rapid relief when compared to traditional therapies for pediatric populations, though the recently expanding body of evidence is smaller than what is available for treatment-resistant unipolar depression [40]. Retrospective studies and reports have shown that intranasal ketamine in treatment-resistant youths (ages 6-19) with bipolar disorders produced significant improvements in mood and behavioral symptoms of mania, alongside being well-tolerated [41]. Studies similarly indicate ketamine can achieve response rates ranging from 52% to 80%, significantly higher than placebo at 5%, in adult patients [42]. Low-dose ketamine has shown tolerability with improving behavioral outcomes such as social withdrawal and hyperactivity in pediatric autism spectrum patients with activity-dependent neuroprotector homeobox (ADNP) gene mutation, despite adverse effects like elation and fatigue [43]. Weekly ketamine dosing reduced Fear Questionnaire (FQ) and Hamilton Anxiety Rating Scale (HAMA) scores by approximately 50% within an hour, and dissociative symptoms diminished over time, with Calgary Depression Scale for Schizophrenia (CADSS) scores declining from 20 in week 1 to 8.8 by week 14 [44]. Despite its benefits, ketamine was associated with transient increases in blood pressure (~10 mmHg, P < 0.05) and nausea in early treatment stages, which were resolved by week 14 [44].

Another study identified that while some hemodynamic changes were reported with ketamine administration, all resolved promptly, and none required any medical intervention [45]. For anxious depression, ketamine provided reductions in MADRS scores in both anxious and non-anxious patients, but baseline MADRS scores were significantly higher in the anxious group (35.3 ± 7.0 vs. 31.4 ± 5.1, P < 0.0001) [46]. Though evidence is early in development, early indicators point to ketamine demonstrating therapeutic efficacy in bipolar depression [16]. These findings underscore ketamine’s rapid onset, broad efficacy, and tolerability across diverse mood disorder populations, including pediatric patients, though its use must be carefully tailored to mitigate side effects. Further research is needed before standardized ketamine treatment protocols can be established in youth and adolescents.

Psilocybin clinical evidence and comparative efficacy: Psilocybin similarly demonstrates the potential for treating mood disorders, with evidence highlighting its rapid and sustained effects on anxiety and depression symptoms of bipolar disorder. In patients with anxiety disorders, PAT significantly reduced symptoms, with 70% of participants experiencing clinical remission at six months post-treatment [10, 13]. The University of Zagreb School of Medicine highlighted a case study of a 16-year-old male patient with debilitating anxiety affecting his school performance and socialization, and who had also failed integration into psychotherapeutic group therapy, garnering significant improvements after orally ingesting 20 to 30 mg psilocybin on three occasions over 18 months [17]. After avoiding group therapy due to his anxiety, he attended and communicated emotionally in a group context after neural activation through psilocybin use [17]. This unique adolescent case report is paralleled by adult studies in which psilocybin has shown promise in facilitating emotional regulation and enhancing neural plasticity, suggesting it could also mitigate both anxiety and depressive symptoms in bipolar depression [2, 16, 19]. More research is needed for bipolar depression to validate its safety; however, as early indications show that classic psychedelics like psilocybin have some association with inducing mania, though it is not the only medicinal therapy associated with such risks (SSRIs) [2, 16, 19]. The therapeutic potential of psilocybin is further supported by its ability to promote introspection and long-lasting shifts in emotional and cognitive frameworks, making it an appealing alternative or adjunct to ketamine for mood disorders [38]. Together, these agents may expand the treatment landscape for pediatric mood disorders, although again further studies are needed in the pediatric psychiatry population. The findings are summarized in Table 4.

**Table 4:** Comparative therapies used for Mood Disorders 5-HT2A: 5-hydroxytryptamine receptor 2A; NMDA: N-methyl-D-aspartate; HR: Heart rate; BP: Blood pressure; ADNP: Activity-dependent neuroprotector homeobox; GAD: Generalized anxiety disorder; MAP: Mean arterial pressure; SUD: Substance use disorder

Author/ Year/ Country	Study Population	Mechanism of Action	Benefits	Adverse Effects	Contraindications/ Caution
Irizarry, 2022, USA [2]	Meta-analysis of adult psychiatric treatment data n=237	Psilocybin: 5-HT2A receptor activation	Broad anxiolytic and antidepressant effects across multiple disorders	Acute reactions with variability in adverse effects due to study design	Patients with a history of psychotic disorders or certain personality disorders, cardio-vascular issues
Bosch, 2022, Switzerland [16]	Case study review of a 21-year-old woman with bipolar disorder	Psilocybin: 5-HT2A receptor activation	Broad antidepressant effects	Induction of psychotic mania	Positive family history of bipolar disorder
Bogadi, 2021, Croatia [17]	Case study of a 16-year-old male with debilitating anxiety	Psilocybin: 5-HT2A receptor activation	Improved socialization and engagement in group psychotherapy	Mild increases in HR and BP, nausea, headache, dizziness	History of psychotic disorders or hallucinations
Papolos, 2013, USA [41]	6–19-year-old patients with bipolar disease n=12	Ketamine: NMDA receptor antagonist	Improvement in mood, anxiety, and behavioral symptoms of mania and aggression with intranasal administration	Dizziness, elated mood state, loss of balance, drowsiness	Untenable medical comorbidities
Bahji, 2021, Canada & USA [42]	Systematic review of adult patients with bipolar depression n=135	Ketamine: NMDA receptor antagonist	Reduction in baseline depression severity	Dissociative symptoms and potential for manic symptom induction (rare)	Comorbid psychosis or current substance addiction, pregnancy
Kolevzon, 2022, USA [43]	6–12-year-old children with ADNP syndrome n=10	Ketamine: NMDA receptor antagonist	Improvements in social behavior, attention deficit and hyperactivity, restricted and repetitive behaviors	Elation, fatigue, increased aggression	Concomitant medication changes within 4 weeks before study enrollment
Glue, 2018, New Zealand [44]	Adult patients with treatment-refractory GAD n=20	Ketamine: NMDA receptor antagonist	Anxiety reduction, functional improvement, subcutaneous administration	Nausea, dizziness, blurred vision, increased MAP	Patients with a SUD due to potential for misuse

Post-Traumatic Stress Disorder (PTSD)

Ketamine clinical evidence and comparative efficacy: Ketamine has emerged as a promising therapy for PTSD in the adult population, offering rapid symptom relief and distinct advantages compared to traditional treatments, though as with mood disorders above, ketamine’s research base in treating child and adolescent PTSD is in its early stages. Ketamine-assisted psychotherapy (KAP) has shown promise in alleviating PTSD, with reductions in mean Post-Traumatic Stress Disorder Checklist for Civilian (PCL-C) scores, alongside supporting self-transcendence [3]. KAP in a 14-year-old adolescent female with PTSD who received sublingual ketamine before sessions, followed by sessions with intramuscular ketamine, achieved a PCL-C score reduction from 51 to 39 after completing KAP, alongside PTSD symptom and functional improvements [47]. Although some have raised concerns about ketamine’s long-term safety, studies have demonstrated that repeated dosing, a facet of treatment that regularly scheduled KAP requires, of up to 42 doses of ketamine over a maximum of four months yielded no serious adverse side effects after pediatric subjects were followed for up to six months after receiving treatment [48]. While PTSD symptom improvements can be seen after only one dose of ketamine, traditional therapies such as SSRIs generally take four to six weeks to exhibit noticeable effects and can take up to 12 weeks to see improvement, which is significant when considering potentially acute psychotherapeutic needs in youth and adolescents [49]. Ketamine has displayed effective PTSD relief in adults, as a proof-of-concept RCT showed that a single intravenous subanesthetic dose of ketamine (0.5 mg/kg) significantly reduced PTSD symptom severity, with a mean difference of 12.7 points on the Impact of Event Scale-Revised (IES-R) (95% CI: 2.5-22.8; P = .02) within 24 hours, alongside improvements in depressive symptoms without persistent dissociative side effects [50]. In a RCT involving six ketamine infusions over two weeks, PTSD symptom severity decreased by an average of 11.88 points on Clinician-Administered PTSD Scale for DSM-V (CAPS-5) scores (SE = 3.96, d = 1.13, 95% CI = 0.36-1.91), with a 67% response rate compared to 20% in the midazolam group, and with benefits persisting for a median of 27.5 days post-treatment [51]. However, outcomes vary across populations; a trial (n=158) in veterans and military personnel showed no significant reduction in PTSD symptoms compared to saline, though significant reductions in depressive symptoms were noted (P < .05) [52]. These findings underscore ketamine’s potential as a rapid and effective PTSD treatment in the pediatric population, while also identifying the need for further comparative research on ketamine’s long-term outcomes in treating youth and adolescent PTSD.

Psilocybin clinical evidence and comparative efficacy: Psilocybin also holds potential for treating PTSD in youth, particularly in its ability to enhance emotional processing and reduce the impact of traumatic memories. Through 5-HT2A receptor activation, psilocybin fosters neural plasticity and connectivity and has allowed adult patients to revisit and reframe traumatic experiences under therapeutic guidance [10, 19]. Compared to ketamine, which provides rapid but transient symptom relief, psilocybin offers longer-lasting effects following a single administration, making it an appealing adjunct or alternative in chronic PTSD cases [13]. Furthermore, psilocybin's ability to facilitate deep emotional breakthroughs and reduce amygdala hyperactivity may complement ketamine's effects, broadening therapeutic possibilities for patients with complex and treatment-resistant PTSD [39]. Additional studies exploring the potential for similar findings in youth with PTSD are the next step to discover psilocybin’s possible therapeutic benefit for this demographic. The findings are summarized in Table 5.

**Table 5:** Comparative therapies used for Post-Traumatic Stress Disorder TRD: Treatment Resistant Depression; PTSD: Post-Traumatic Stress Disorder; KAP: Ketamine-assisted psychotherapy; NMDA: N-methyl-D-aspartate; SSRI: Selective Serotonin Reuptake Inhibitor; IES-R: Impact of Event Scale-Revised; BP: Blood pressure; HR: Heart rate; MARDS: Montgomery–Åsberg Depression Rating Scale

Author/ Year/ Country	Study Population	Mechanism of Action	Benefits	Adverse Effects	Contraindications/ Caution
Montjoy, 2022, USA [3]	Adults with TRD or PTSD n=33	KAP: Psycho-therapeutic intervention with NMDA receptor antagonism	PTSD and depression symptom reduction, self-transcendence	Dissociation	Further research needed
Wolfson, 2023, USA [47]	Case report of 14 y/o female with PTSD	KAP: Psycho-therapeutic intervention with NMDA receptor antagonism	Decrease in PTSD checklist/civilian scores, PTSD symptom, and functional improvements	Nausea	Evidence of thought disorders, hallucinations, or delusions
James, 2023, England [48]	5–18-year-old pediatric patients n=87	Ketamine: NMDA receptor antagonist	Safety of repeat dosing, antidepressant effects and potential for linked PTSD symptom reduction	No serious adverse events nor long-term consequences after up to 6 months follow-up	Hypothetical cautions regarding long-term safety for children’s developing brains
Lang, 2024, USA [49]	Meta-analysis of veterans with PTSD	SSRIs: selective serotonin reuptake inhibitors	Effective treatments for those who cannot undergo psychotherapy	Gastrointestinal disturbances, headache, delayed onset of therapeutic effects	Avoid in patients with bipolar disorder to avoid inducing manic episodes
Feder, 2014, USA [50]	Adult patients with chronic PTSD n=41	Ketamine: NMDA receptor antagonist	Rapid PTSD symptom severity with improved IES-R scores	Increase in BP and HR, perceptual disturbance, dissociative symptoms	History of psychotic or bipolar disorder, current bulimia or anorexia nervosa, recent alcohol abuse or dependence
Feder, 2021, USA [51]	Adult patients with chronic PTSD n=30	Ketamine: NMDA receptor antagonist	Significant reduction in PTSD symptom severity, rapid onset	Dissociative symptoms, dizziness, and elevated BP during infusions	Uncontrolled hypertension, history of psychotic disorders, recent substance abuse
Abdallah, 2022, USA [52]	Veterans and service members with PTSD n=158	Ketamine: NMDA receptor antagonist	Reduced MARDS scores, though no dose-related reduction in PTSD symptoms	Transient dissociative and psychoto-mimetic effects	Psychotic disorder or features, manic or mixed episodes, unstable medical condition, severe brain injury

Ethical considerations

In considering the therapeutic efficacy of the dissociative ketamine and the hallucinogenic psilocybin in the pediatric population, ethical questions have been raised in the medical community regarding these emerging therapies’ use. Alongside the above current state evaluation, a brief overview of some of the main ethical issues posed in the literature warrants exploration. Our goal is not to provide exhaustive arguments, but to use value-neutral language to bring light to these broader discussions for the sake of awareness. During the drug revolution of the 1960s and 1970s, researchers investigating the physiological effects of psychedelics in youth and adolescents did not promote their use, and many states made use of these substances illegal [53]. While some currently believe this evidence is sufficiently robust for those decisions to stand, others identified methodological limitations in this past research, consequently leaving a fresh opportunity to examine their use in the present day [54]. First, the use of dissociative or psychedelic drugs in patients who are experiencing hallucinations or other symptoms of psychosis raises questions regarding their suitability for supporting reality orientation, which contributed to why we chose to examine TRD, mood disorders, and PTSD, none of which have psychosis necessarily connected to their diagnostic criteria.

Second, the use of psychedelics in the pediatric population has raised questions about the potential for durable changes in youth’s brains [54]. Existing psychotropic therapies like anxiolytics, antidepressants, and psychotherapy have demonstrated neuroplasticity alterations gradually over time, while psychedelic-assisted therapy can make quick neuroplastic changes with potentially long-lasting impacts on mood and personality [10, 54]. Limited research exists on psilocybin’s long-term impact on children’s developing brains, raising concerns about the risks of neurotoxicity or altered neural connectivity [14, 39]. Additionally, pediatric patients with pre-existing mental health conditions may respond unpredictably to psilocybin, with potential risks of adverse psychological reactions, such as panic, dissociation, or psychosis [55]. For ketamine, relatively little is known regarding its longer-term effects on the developing brains of youths, and if there is an increased risk for dependence or abuse [56]. Do the benefits outweigh the risks? Further long-term research on ketamine and psilocybin in the adolescent population, alongside the development of therapy protocols first in the adult population, is needed before they can become standardized treatments in pediatrics [57].

Third, questions have been raised about whether parental consent for use of dissociative and/or hallucinogenic drugs must also coincide with consent from the youth or adolescent receiving treatment [58]. For ketamine, one study identified parents had high acceptability towards its use for children with suicidality, MDD, and bipolar disorder, although some others had concerns about adverse side effects and why it is not FDA approved [59]. In this vein, other issues regarding informed consent and children’s capacity to fully understand the risks and implications of such treatment have been highlighted in the research [60]. To what degree does family history play into the risk evaluation of ketamine and psilocybin use? Currently, age-appropriate evaluation criteria for use of dissociatives or psychedelics in those with a family history of substance use disorder, or other predisposing trauma, remain undeveloped [54]. Finally, establishing legal frameworks for these therapeutic agents will be important before the creation of new treatment settings [16]. Therefore, rigorous safety protocols, professional supervision, and thorough preclinical research are essential to have established when considering ketamine and psilocybin as therapeutic options in pediatric populations. With these in place, the potential for these treatments to provide therapeutic benefits to pediatric patients in need can be safely utilized.

## Conclusions

Both ketamine and psilocybin are emerging as therapeutic options worthy of consideration for use in pediatric TRD, mood disorders, and PTSD when compared to conventional therapies. Observed increases in the use of ketamine as an anesthetic in pediatric patients give credence to further evaluation of its multifaceted indications, like in pediatric psychiatry. Our findings highlighted ketamine’s rapid-onset, clinically significant, and sustained antidepressant impact, with manageable adverse effects, which positions it as a novel treatment option in managing pediatric TRD. Subanesthetic doses of ketamine have likewise shown symptom improvements in pediatric mood disorders such as anxiety and bipolar depression. For PTSD, ketamine has displayed potential for broad symptom relief when compared to SSRIs for intrusive memories and emotional dysregulation, especially when combined with psychotherapy. Ketamine could fill a clinically urgent gap in rapid-acting treatments for pediatric mental health disorders left by slower and possibly less effective conventional therapies, may represent a significant potential treatment in the management of complex conditions, and may potentially be utilized in clinical settings that lend towards quick therapy interventions via various routes of administration, such as emergency rooms.

Psilocybin, like ketamine, has therapeutic potential for complex and treatment-resistant psychiatric conditions in the pediatric population. Its unique mechanism, targeting 5-HT2A receptors and promoting neuroplasticity, has enabled profound and lasting shifts in emotional processing for mood disorders and PTSD in adults. Recent studies have shown that PAT can achieve clinical remission in conditions like TRD and PTSD, with positive effects lasting for months after a single dose. A case study of psilocybin’s significant impact on the socialization and psychotherapeutic progress of an adolescent with debilitating anxiety points to an opportunity for further study of psilocybin’s potential in this population. Psilocybin's capacity for fostering introspection and emotional breakthrough complements ketamine's rapid-onset symptom relief, creating opportunities for exploring integrative treatment approaches that leverage the strengths of both agents for youth and adolescents. While there are ethical considerations implicated in the use of dissociative and hallucinogenic therapies in the pediatric population, if appropriate ethical and legal criteria can be established for such use, ketamine and psilocybin could represent new frontiers in pediatric psychiatry, offering hope to patients for whom traditional treatments have been unsuccessful.

## References

[1] Kim S, Rush BS, Rice TR (2021). A systematic review of therapeutic ketamine use in children and adolescents with treatment-resistant mood disorders. Eur Child Adolesc Psychiatry.

[2] Irizarry R, Winczura A, Dimassi O, Dhillon N, Minhas A, Larice J (2022). Psilocybin as a treatment for psychiatric illness: a meta-analysis. Cureus.

[3] Montjoy JF (2022). Ketamine-Assisted Psychotherapy: Clinical Outcomes and Self-Transcendence in Depression and Post-Traumatic Stress Disorder [dissertation]. Tucson, AZ: University of Arizona.

[4] Rawat R, Tunc-Ozcan E, Dunlop S (2024). Ketamine's rapid and sustained antidepressant effects are driven by distinct mechanisms. Cell Mol Life Sci.

[5] Bali A, Dang AK, Gonzalez DA, Kumar R, Asif S (2022). Clinical uses of ketamine in children: a narrative review. Cureus.

[6] Li CT, Yang KC, Lin WC (2018). Glutamatergic dysfunction and glutamatergic compounds for major psychiatric disorders: evidence from clinical neuroimaging studies. Front Psychiatry.

[7] Averill LA, Purohit P, Averill CL, Boesl MA, Krystal JH, Abdallah CG (2017). Glutamate dysregulation and glutamatergic therapeutics for PTSD: evidence from human studies. Neurosci Lett.

[8] Dolansky G, Shah A, Mosdossy G, Rieder M (2008). What is the evidence for the safety and efficacy of using ketamine in children?. Paediatr Child Health.

[9] Alexander L, Young AH (2023). Recent advances in the psychopharmacology of major depressive disorder. BJPsych Adv.

[10] Barber GS, Aaronson ST (2022). The emerging field of psychedelic psychotherapy. Curr Psychiatry Rep.

[11] Dwyer JB, Landeros-Weisenberger A, Johnson JA (2021). Efficacy of intravenous ketamine in adolescent treatment-resistant depression: a randomized midazolam-controlled trial. Am J Psychiatry.

[12] Ng KT, Sarode D, Lai YS, Teoh WY, Wang CY (2019). The effect of ketamine on emergence agitation in children: a systematic review and meta-analysis. Paediatr Anaesth.

[13] Davis AK, Barrett FS, May DG (2021). Effects of psilocybin-assisted therapy on major depressive disorder: a randomized clinical trial. JAMA Psychiatry.

[14] Johnson MW, Griffiths RR (2017). Potential therapeutic effects of psilocybin. Neurotherapeutics.

[15] Goodwin GM, Aaronson ST, Alvarez O (2022). Single-dose psilocybin for a treatment-resistant episode of major depression. N Engl J Med.

[16] Bosch OG, Halm S, Seifritz E (2022). Psychedelics in the treatment of unipolar and bipolar depression. Int J Bipolar Disord.

[17] Bogadi M, Kaštelan S (2021). A potential effect of psilocybin on anxiety in neurotic personality structures in adolescents. Croat Med J.

[18] Goel DB, Zilate S (2022). Potential therapeutic effects of psilocybin: a systematic review. Cureus.

[19] Wojtas A, Bysiek A, Wawrzczak-Bargiela A (2022). Effect of psilocybin and ketamine on brain neurotransmitters, glutamate receptors, DNA and rat behavior. Int J Mol Sci.

[20] Nichols DE (2016). Psychedelics. Pharmacol Rev.

[21] Millan MJ, Marin P, Bockaert J, Mannoury la Cour C (2008). Signaling at G-protein-coupled serotonin receptors: recent advances and future research directions. Trends Pharmacol Sci.

[22] Barnes NM, Sharp T (1999). A review of central 5-HT receptors and their function. Neuropharmacology.

[23] Matias S, Lottem E, Dugué GP, Mainen ZF (2017). Activity patterns of serotonin neurons underlying cognitive flexibility. Elife.

[24] Page MJ, McKenzie JE, Bossuyt PM (2021). The PRISMA 2020 statement: an updated guideline for reporting systematic reviews. BMJ.

[25] Herd DW, Anderson BJ, Keene NA, Holford NH (2008). Investigating the pharmacodynamics of ketamine in children. Paediatr Anaesth.

[26] Patel N (2013). Comparison of cardiovascular and respiratory changes during induction, maintenance and recovery with sevoflurane and propofol in pediatric day care anesthesia. J Chem Pharm Res.

[27] Zhao Y, Qin F, Liu Y, Dai Y, Cen X (2022). The safety of propofol versus sevoflurane for general anesthesia in children: a meta-analysis of randomized controlled trials. Front Surg.

[28] Ibrahim ME, Nahar N, Ferdous A, Loban AH (2023). Low dose propofol at the end of sevoflurane anesthesia reduces emergence agitation in children: a prospective observational study. Saudi J Med Pharm Sci.

[29] Mansuri Z, Shah B, Yadav G (2024). Is intravenous ketamine better than intranasal esketamine for treating treatment-resistant depression?. Prim Care Companion CNS Disord.

[30] Zhou Y, Lan X, Wang C (2024). Effect of repeated intravenous esketamine on adolescents with major depressive disorder and suicidal ideation: a randomized active-placebo-controlled trial. J Am Acad Child Adolesc Psychiatry.

[31] Meshkat S, Rosenblat JD, Ho RC (2022). Ketamine use in pediatric depression: a systematic review. Psychiatry Res.

[32] Bahji A, Vazquez GH, Zarate CA Jr (2021). Comparative efficacy of racemic ketamine and esketamine for depression: a systematic review and meta-analysis. J Affect Disord.

[33] Loo C, Glozier N, Barton D (2023). Efficacy and safety of a 4-week course of repeated subcutaneous ketamine injections for treatment-resistant depression (KADS study): randomised double-blind active-controlled trial. Br J Psychiatry.

[34] Cullen KR, Amatya P, Roback MG (2018). Intravenous ketamine for adolescents with treatment-resistant depression: an open-label study. J Child Adolesc Psychopharmacol.

[35] Kishimoto T, Chawla JM, Hagi K, Zarate CA, Kane JM, Bauer M, Correll CU (2016). Single-dose infusion ketamine and non-ketamine N-methyl-d-aspartate receptor antagonists for unipolar and bipolar depression: a meta-analysis of efficacy, safety and time trajectories. Psychol Med.

[36] Kang MJ, Hawken E, Vazquez GH (2022). The mechanisms behind rapid antidepressant effects of ketamine: a systematic review with a focus on molecular neuroplasticity. Front Psychiatry.

[37] MacCallum CA, Lo LA, Pistawka CA, Deol JK (2022). Therapeutic use of psilocybin: practical considerations for dosing and administration. Front Psychiatry.

[38] Carhart-Harris RL, Goodwin GM (2017). The therapeutic potential of psychedelic drugs: past, present, and future. Neuropsychopharmacology.

[39] Carhart-Harris RL, Roseman L, Bolstridge M (2017). Psilocybin for treatment-resistant depression: fMRI-measured brain mechanisms. Sci Rep.

[40] McCloud TL, Caddy C, Jochim J (2015). Ketamine and other glutamate receptor modulators for depression in bipolar disorder in adults. Cochrane Database Syst Rev.

[41] Papolos DF, Teicher MH, Faedda GL, Murphy P, Mattis S (2013). Clinical experience using intranasal ketamine in the treatment of pediatric bipolar disorder/fear of harm phenotype. J Affect Disord.

[42] Bahji A, Zarate CA, Vazquez GH (2021). Ketamine for bipolar depression: a systematic review. Int J Neuropsychopharmacol.

[43] Kolevzon A, Levy T, Barkley S (2022). An open-label study evaluating the safety, behavioral, and electrophysiological outcomes of low-dose ketamine in children with ADNP syndrome. HGG Adv.

[44] Glue P, Neehoff SM, Medlicott NJ, Gray A, Kibby G, McNaughton N (2018). Safety and efficacy of maintenance ketamine treatment in patients with treatment-refractory generalised anxiety and social anxiety disorders. J Psychopharmacol.

[45] Bruton AM, Wesemann DG, Machingo TA, Majak G, Johnstone JM, Marshall RD (2025). Ketamine for mood disorders, anxiety, and suicidality in children and adolescents: a systematic review. Eur Child Adolesc Psychiatry.

[46] Salloum NC, Fava M, Freeman MP (2019). Efficacy of intravenous ketamine treatment in anxious versus nonanxious unipolar treatment-resistant depression. Depress Anxiety.

[47] Wolfson PE, Andries J, Ahlers D, Whippo M (2023). Ketamine-assisted psychotherapy in adolescents with multiple psychiatric diagnoses. Front Psychiatry.

[48] James L, James IG, Wakefield J (2023). The safety of repeated ketamine dosing in paediatrics: a systematic review. J Psychopharmacol.

[49] Lang AJ, Hamblen JL, Holtzheimer P (2024). A clinician's guide to the 2023 VA/DoD Clinical Practice Guideline for Management of Posttraumatic Stress Disorder and Acute Stress Disorder. J Trauma Stress.

[50] Feder A, Parides MK, Murrough JW (2014). Efficacy of intravenous ketamine for treatment of chronic posttraumatic stress disorder: a randomized clinical trial. JAMA Psychiatry.

[51] Feder A, Costi S, Rutter SB (2021). A randomized controlled trial of repeated ketamine administration for chronic posttraumatic stress disorder. Am J Psychiatry.

[52] Abdallah CG, Roache JD, Gueorguieva R (2022). Dose-related effects of ketamine for antidepressant-resistant symptoms of posttraumatic stress disorder in veterans and active duty military: a double-blind, randomized, placebo-controlled multi-center clinical trial. Neuropsychopharmacology.

[53] Waldman E, Adrian C, Spence D (2023). Psychedelic-assisted therapies and pediatric palliative care: new tools for hope and healing (TH118A). J Pain Symptom Manage.

[54] Edelsohn GA, Sisti D (2023). Past is prologue: ethical issues in pediatric psychedelics research and treatment. Perspect Biol Med.

[55] Rucker JJ, Iliff J, Nutt DJ (2018). Psychiatry & the psychedelic drugs. Past, present & future. Neuropharmacology.

[56] Yavi M, Lee H, Henter ID, Park LT, Zarate CA Jr (2022). Ketamine treatment for depression: a review. Discov Ment Health.

[57] Vorobyeva N, Kozlova AA (2022). Three naturally-occurring psychedelics and their significance in the treatment of mental health disorders. Front Pharmacol.

[58] Rajwani K (2022). Should adolescents be included in emerging psychedelic research?. Can J Bioeth Rev.

[59] Mathai DS, McCathern AG, Guzick AG (2021). Parental attitudes toward use of ketamine in adolescent mood disorders and suicidality. J Child Adolesc Psychopharmacol.

[60] Hendricks PS, Johnson MW, Griffiths RR (2015). Psilocybin, psychological distress, and suicidality. J Psychopharmacol.

